# Mapping evidence on tuberculosis active case finding policies, strategies, and interventions for tuberculosis key populations: a systematic scoping review protocol

**DOI:** 10.1186/s13643-019-1098-1

**Published:** 2019-07-10

**Authors:** Desmond Kuupiel, Vitalis Bawontuo, Tivani P. Mashamba-Thompson

**Affiliations:** 1 Discipline of Public Health Medicine, Department of Public Health Medicine, School of Nursing and Public Health, University of KwaZulu-Natal, 2nd Floor George Campbell Building, Howard College Campus, Durban, 4001 South Africa; 2 Research for Sustainable Development Consult, Sunyani, Ghana; 3 Faculty of Health and Allied Sciences, Catholic University College of Ghana, Fiapre, Sunyani, Ghana

**Keywords:** Tuberculosis, Active case finding, Tuberculosis key populations

## Abstract

**Background:**

Early diagnosis and treatment of tuberculosis (TB) have been shown to reduce the impact of TB illness particularly, among TB key populations such as people living with HIV/AIDS, prisoners, refugees, migrants, displaced populations, survivors of TB illness, and indigenous populations. We propose to conduct a systematic scoping review to map global evidence on active tuberculosis case finding policies, strategies, and interventions for TB key populations.

**Method and analysis:**

This study will be guided by the scoping review framework, proposed by Arksey and O’Malley. A comprehensive literature search will be performed in the following electronic databases: PubMed, SCOPUS, Web of Science, Science Direct, and EBSCOhost (Academic search complete, PsycINFO, Health Sources, CINAHL, and MEDLINE with full text). Primary studies both published in peer-reviewed journals and grey literature such as unpublished studies, thesis, and studies in press addressing our research question will be included. To reduce selection bias, two independent reviewers will perform title, abstract, and full article screening in parallel. Also, data extraction from the included studies will be conducted by two independent reviewers. We will use NVivo version 11 software to extract the outcomes relevant to answering the research question from the included studies using a content thematic analysis. The results of this proposed study will be presented according to the Preferred Reporting Items for Systematic Reviews and Meta-Analysis: Extension for Scoping Review (PRISMA-ScR). The mixed methods assessment tool version 2018 will be employed for quality appraisal of the included studies.

**Discussion:**

We anticipate that the mapped evidence will help reveal diverse active TB case finding policies, strategies, and interventions to help inform future adoption and implementation to reduce TB missing cases worldwide. We also anticipated that the results of the proposed scoping review will help reveal research gaps, which can be addressed to ensure early detection and treatment of TB among key populations. The proposed study will may as well contribute to healthcare systems strengthening and improve research in TB key populations.

## Background

Tuberculosis (TB) is a poverty-related disease and the world’s leading killer among infectious diseases [1, 2]. Worldwide, almost 1.5 million people die of TB illness each year making it one of the top ten causes of death [1, 3, 4] with over 94% of all TB deaths occurring in low- and middle-income countries yearly [1, 4]. Globally, close to a third of people have latent TB infection and places them at risk of developing TB illness [1]. However, people living with human immune-deficiency virus (HIV) infection or acquired immuno-deficiency syndrome (AIDS), prisoners, migrants, refugees, survivors of TB infection, and indigenous populations are highly vulnerable and at much higher risk of developing TB illness [3].

Of the 10 million people fell who ill with TB globally in 2017, nearly 30% of them were “missing” that is, people with TB who did not report, undiagnosed, or untreated [3, 5–7]. TB key populations such as prisoners, people living with TB/HIV coinfection, migrants, refugees, and indigenous populations may have poor health-seeking behaviour and experience significant marginalisation and human rights violations [3, 8]. They also experience far-reaching stigma and discrimination, state and non-state violence and harassment, restrictive laws and policies, and criminalization of behaviours or practices which put them at higher risks and also undermine their access to quality healthcare services [3, 8–10]. TB is spread from person to person through coughing and sneezing [11, 12]. It is estimated that one person with active or untreated TB can spread the infection to as many as 15 other people in a year. This makes the hunt for “missing cases” more urgent [3, 13].

To address the challenge of TB “missing cases”, policies, effective strategies, and implementation of active case interventions for TB key populations are highly essential globally [14, 15]. Active TB case finding approaches such as screening of TB contacts, workplace screening, screening prior to entry into a therapy or drug treatment, mandatory screening in the criminal justice system, and refugee programmes are essential to facilitate early detection of TB case and treatment. This study therefore aims to map global evidence on active tuberculosis case finding policies, strategies, and interventions for TB key populations. We anticipate that the results of this study will reveal research gaps for future meta-analysis or primary studies to inform policy towards ending TB epidemics as stipulated in end TB strategy [16].

## Methods

### Overview

A scoping review is useful in mapping a range of literature that exists around a topic of interest and aids to focus the research questions by charting existing research findings and identifying research gaps [17]. A scoping methodology is also considered a useful approach for determining the need and value of a future primary study or a full systematic review [17]. Based on this, the current scoping review will be guided by the enhanced Arksey and O’Malley scoping review framework [17, 18]. The framework involves the following: identifying the research question, identifying relevant studies, study selection, charting the data, and collating, summarising, and reporting results.

### Identifying the research question

Population, Concept and Context (PCC) mnemonic [19] was used to determine the eligibility of the scoping review question, as shown in Table 1. The main review question will be What evidence exist on active TB case finding policies, strategies, and interventions for TB key populations globally?

**Table 1:** PCC framework for defining the eligibility of the scoping review question

P-Population	TB key populations (prisoners, people living with HIV, refugees, survivors of TB illness, migrants, and indigenous populations)
C-Concept	Active TB case finding (A strategy whereby healthcare providers or researchers actively go to search for new TB cases among key populations in his/her community or study setting
C-Context	Global policies, strategies, and interventions

The sub-review questions will be as follows:

- What evidence exist on polices for active TB case finding in key populations globally?
- What evidence exist on strategies for active TB case finding in key populations globally?
- What evidence exist on active TB case finding interventions for key populations globally?

### Identify relevant studies

We will conduct a thorough keyword search to identify all relevant studies from multiple databases regardless of the date of publication, language, and publication status (published, unpublished, and in press). We will search the following electronic databases: PubMed, SCOPUS, Web of Science, Science Direct, and EBSCOhost (Academic Search Complete, PsycINFO, Health Sources, CINAHL, and MEDLINE with full text) for relevant studies. The reference list of all included studies will also be thoroughly for relevant articles. The combination of keywords that will be used to search for relevant studies from the electronic databases are “active tuberculosis”, “case finding”, “policy”, “intervention”, “strategies”, “key population” “HIV patients”, “prisoners”, “refugees”, “indigenous populations”, “migrants”, “survivor of tuberculosis illness”, and “hard-to-reach populations”. Boolean terms, AND/OR, and Medical Subject Heading (MeSH) terms will be used during the keywords search to identify relevant studies. Details of the search records such as the date of the search, database, keywords, number of studies, and number of eligible studies will be adequately documented for each search as demonstrated for the pilot search in PubMed (Table 2).

**Table 2:** Pilot search in PubMed electronic database

Date	Database	Keywords	Search results
10/06/2019	PubMed	“tuberculosis”[All Fields]) OR TB[All Fields]) OR (“tuberculosis”[MeSH Terms] OR “tuberculosis”[All Fields] OR (“koch’s”[All Fields] AND “disease”[All Fields]) OR “koch’s disease”[All Fields])) AND ((“IEEE Int Conf Automation Sci Eng (CASE)”[Journal] OR “CASE (Phila)”[Journal] OR “case”[All Fields]) AND (“signs and symptoms”[MeSH Terms] OR (“signs”[All Fields] AND “symptoms”[All Fields]) OR “signs and symptoms”[All Fields] OR “finding”[All Fields]))) OR (active[All Fields] AND (“IEEE Int Conf Automation Sci Eng (CASE)”[Journal] OR “CASE (Phila)”[Journal] OR “case”[All Fields]) AND (“signs and symptoms”[MeSH Terms] OR (“signs”[All Fields] AND “symptoms”[All Fields]) OR “signs and symptoms”[All Fields] OR “finding”[All Fields])) OR (“epidemiology”[Subheading] OR “epidemiology”[All Fields] OR “surveillance”[All Fields] OR “epidemiology”[MeSH Terms] OR “surveillance”[All Fields])	2418156

### Eligibility criteria

To ensure the selection of relevant studies for this review, the study selection will be guided by the eligibility criteria as specified under the inclusion/exclusion criteria.

#### Inclusion criteria

We will include studies that meet the following criteria:

- Studies presenting evidence on the study conducted among TB key populations globally
- Studies reporting evidence on active case finding policies for TB key populations globally
- Studies reporting evidence on active case finding strategies for TB key populations globally
- Studies presenting evidence on active case finding intervention for TB key populations globally
- Quantitative, qualitative, and mixed methods study designs

#### Exclusion criteria

This will include the following:

- Studies targeting the general population
- Studies focusing on health education on TB
- Qualitative study designs
- Other types of reviews
- Studies reporting on diagnostic accuracy without yield

### Study selection

To reduce selection bias, two independent investigators will conduct a comprehensive title screening in the abovementioned electronic databases guided by the eligibility criteria. All eligible studies will be imported into endnote X9 software and duplicates removed. The final endnote library will then be shared among the reviewers. Two investigators will also independently sort the studies into the categories “excluded” and “included” at the abstract and full-text screening stages, based on the inclusion criteria listed above. In an event where an article could not be retrieved from the databases, we will request for assistance from the University of KwaZulu-Natal library services or we may request for the full text from the authors. Categorisation choices will be compared and, where there is disagreement, the researchers will discuss the title and content of the study until a consensus is reached on a categorisation during the title and abstract screening stages. A third investigator will be engaged to resolve disagreement at the full-text screening stage. We will also calculate the inter-rater agreement (Cohen’s kappa coefficient (*κ*) statistic) and between reviewers following full-text screening as well as, the McNemar’s chi-square statistic using Stata 14. For articles with no available abstract, categorisation based on the title alone will be used. We will follow an adapted PRISMA (Preferred Reporting Items for Systematic Reviews and Meta-Analyses) guidelines to report the screening results [20].

### Charting the data

We will extract information relevant to the aim of this study. A data extraction form will be developed electronically using google forms and piloted with ten percent of the included studies by two investigators in order to ensure accuracy and consistency extracted data. The data extraction form will then be updated based on feedback from the investigators if needed before its final use. Table 3 shows the data extraction form that will be used for this proposed review.

**Table 3:** Data extraction form

Author and year of publication	
Study title	
Study aim/objective	
Study setting (country)	
Type of TB key population	
Active case finding policy	
Active case finding strategy	
Active case finding interventions	
Most significant findings	
Study funding information	

### Collating, summarising, and reporting the results

Content thematic analysis approach will be employed to abstract data relevant to answer the proposed scoping review question [21]. All data regarding active TB case finding interventions and strategies for TB key populations published globally will be extracted from the included articles. NVivo version 11 will be used for content thematic analysis of the included studies. The emerging themes from the included studies will be reported by giving a narrative summary of the results to answer the review question. The implications of the study results for future research, policy, and practice will also be examined and reported. The results of this proposed study will be presented according to the Preferred Reporting Items for Systematic Reviews and Meta-Analysis: Extension for Scoping Review (PRISMA-ScR) [20].

### Quality appraisal

To assess the quality of the included studies, the Mixed Method Quality Appraisal Tool (MMAT) Version 2018 [22] will be used to assess the methodological quality of all the included studies. We will use the MMAT to examine the included studies in the following categories: the appropriateness of the aim of the study, adequacy and methodology, study design, participant recruitment, data collection, data analysis, and findings presented. We will grade the quality of studies with a quality score ranging from ≤ 50% as low quality, 51–75% will be considered as an average quality, and 76–100% will be considered as high quality. This will ensure that the study designs of the included studies are appropriate for the research objectives. The quality assessment will also help us to report on the risk of bias of the included studies, and the quality of the evidence that will be reported.

## Discussion

This systematic scoping review will map existing literature on active tuberculosis case finding policies, strategies, and interventions for TB key populations globally. The World Health Organization End TB Strategy aims to end the global TB epidemic, with targets to reduce TB deaths by 95% and to cut new cases by 90% between 2015 and 2035, and to ensure that no family is burdened with catastrophic expenses due to TB [23]. The WHO has also set interim milestones for 2020, 2025, and 2030 [23]. The End Strategy reinforces a focus on serving populations highly vulnerable to TB infection and poor healthcare access, such as hard-to-reach populations [23]. Populations such as people living with HIV/AIDS, prisoners, migrants, refugees, displaced persons, survivors of TB infection, and indigenous populations have been identified as key populations with a higher risk of developing TB illness [3].

We anticipate that the mapped evidence will help reveal diverse active TB case finding policies, strategies, and interventions to help inform future adoption and implementation to reduce TB missing cases worldwide. We also anticipated that the results of the proposed scoping review will help reveal research gaps, which can be addressed to ensure early detection and treatment of TB among key populations. The proposed study will may as well contribute to healthcare systems strengthening and improve research in TB key populations. The study further intends to build and contribute to a body of literature on TB research, particularly on the diagnosis of the most vulnerable populations. Moreover, the results of the proposed scoping review may contribute to eliminating TB by the year 2035.

## Conclusion

The findings of this proposed systematic scoping review will provide evidence useful to influence the direction of future research such as meta-analysis and primary studies to influence policy, strategy, and implementation of active case finding interventions for TB key populations in countries most needed.

## Data Availability

We have duly cited all studies, and data is presented in a form of references.

## Abbreviation

TB: Tuberculosis
